# The role of post-translational modifications in osteonecrosis of the femoral head

**DOI:** 10.3389/fcell.2026.1885721

**Published:** 2026-08-17

**Authors:** Longxin An, Lizhi Cao, Jie Zhao, Xiaoming Yang, Haoyong Ma, Zhongzheng Shi, Zilong Deng, Zhen Liu, Naibo Feng, Xuecheng Sun

**Affiliations:** 1 Department of Trauma Orthopedics, Weifang People’s Hospital, Shandong Second Medical University, Weifang, China; 2 School of Clinical Medicine, Shandong Second Medical University, Weifang, China; 3 Department of Neurology, Weifang People’s Hospital, Shandong Second Medical University, Weifang, China; 4 Yanbian University Hospital, Yanbian University, Yanji, China

**Keywords:** BMSC, endothelial dysfunction, ferroptosis, osteoblast, osteoclast, osteonecrosis of the femoral head, phosphorylation, post-translational modifications

## Abstract

**Background:**

Osteonecrosis of the femoral head (ONFH) is a progressive and disabling orthopedic disorder characterized by impaired bone remodeling and microvascular dysfunction. Emerging evidence identifies post-translational modifications (PTMs) as critical molecular switches linking external pathogenic stimuli, such as glucocorticoids and alcohol, to intracellular signaling dysregulation in ONFH.

**Main Body:**

This review comprehensively summarizes the regulatory roles of major PTMs-including phosphorylation, acetylation, methylation, and ubiquitination-in key cell types governing femoral head homeostasis, namely, bone marrow-derived mesenchymal stem cells, osteoblasts, osteocytes, osteoclasts, and vascular endothelial cells. Aberrant PTMs disrupt the osteogenic-adipogenic balance, impair angiogenesis, and trigger multiple forms of programmed cell death, including apoptosis and ferroptosis, collectively driving the pathological progression towards bone necrosis. Particular emphasis is placed on phosphorylation-dependent signaling pathways (e.g., PI3K/Akt, GSK-3β/β -catenin, JAK2/STAT3, and AMPK/mTOR), ubiquitin-mediated mitochondrial quality control, and acetylation-driven epigenetic regulation. Furthermore, we highlight emerging PTM-targeted small-molecule interventions that show promise in restoring osteogenesis, preserving endothelial function, or suppressing cell death.

**Conclusion:**

Future research directions include decoding cell type-specific “PTM codes”, integrating multi-omics analyses, and developing patient-derived organoid models. Advancing these areas may collectively enable earlier diagnosis and more precise, mechanism-based therapies for ONFH.

## 1 Introduction

Osteonecrosis of the femoral head (ONFH) is a refractory orthopedic disorder characterized by progressive necrosis of bone cells and bone marrow tissues resulting from compromised blood supply to the femoral head. This pathological process ultimately leads to structural collapse of the femoral head and hip joint dysfunction, severely impairing patients’ quality of life (Li Z. et al., 2017). ONFH is generally classified into traumatic and non-traumatic types. Traumatic ONFH arises directly from mechanical injury, such as femoral neck fractures or hip dislocation, whereas non-traumatic ONFH is etiologically complex and involves multiple interacting risk factors (Cheng et al., 2025; Pang et al., 2025; Yin et al., 2025). Alcohol abuse, circulatory dysfunction, long-term glucocorticoid therapy, and congenital hip dysplasia have all been identified as major contributors to the development of non-traumatic ONFH (Pang et al., 2025; Li L. et al., 2024; Xia et al., 2023; Ye et al., 2025; Zheng et al., 2024).

ONFH is a progressive and destructive disease; without timely intervention, it inevitably progresses to femoral head collapse (Zhao et al., 2016; Ikemura et al., 2016). It represents one of the leading causes of hip disability in young and middle-aged adults aged 20–50 years, with approximately 70% of patients ultimately requiring total hip arthroplasty (THA) (Yang et al., 2015). Given the relatively young age and high activity level of this patient population, multiple revision surgeries may be required over a lifetime. However, repeated revision arthroplasty is associated with increased technical difficulty, higher complication rates, and substantial physical and psychological burden (Ma and Zhao, 2020). Therefore, elucidating the molecular mechanisms underlying ONFH, identifying early diagnostic biomarkers, and discovering precise therapeutic targets are of substantial clinical importance and social significance.

Post-translational modifications (PTMs) refer to reversible or irreversible chemical modifications that occur on specific amino acid residues of proteins after translation. By altering protein charge, conformation, intermolecular interactions, and subcellular localization, PTMs precisely regulate protein stability, enzymatic activity, and signal transduction. These modifications play central roles in physiological processes such as embryonic development and tissue homeostasis, as well as in pathological conditions including cancer, cardiovascular diseases, and metabolic disorders (Haro et al., 2022; Zhong et al., 2023). Accumulating evidence demonstrates that PTMs modulate neural development and synaptic plasticity in the nervous system, while in the respiratory and cardiovascular systems they participate in the regulation of mitochondrial homeostasis, myocardial contraction, and vascular tone (Tao J. et al., 2024; Dasgupta et al., 2020; Huang TH. et al., 2024; Mohamed et al., 2021; Cheng et al., 2023; Hong FF. et al., 2019). In the digestive and renal systems, PTMs govern enzyme activation, metabolic balance, and filtration barrier integrity; in hematologic and endocrine systems, they influence coagulation, immune responses, and hormone signaling (Hur et al., 2020; Yun et al., 2016; Troisi et al., 2023; Loh and Su, 2016; Zhang et al., 2021; Yu et al., 2022; Oikawa et al., 2020). Within the musculoskeletal system, PTMs are essential for maintaining bone remodeling and skeletal muscle energy metabolism (Kim et al., 2019; Guhathakurta et al., 2018). Moreover, histone modifications, a core form of epigenetic regulation, have been extensively implicated in tumor initiation and progression, providing promising targets for disease prevention and therapy (Duan et al., 2024).

**FIGURE 1:**
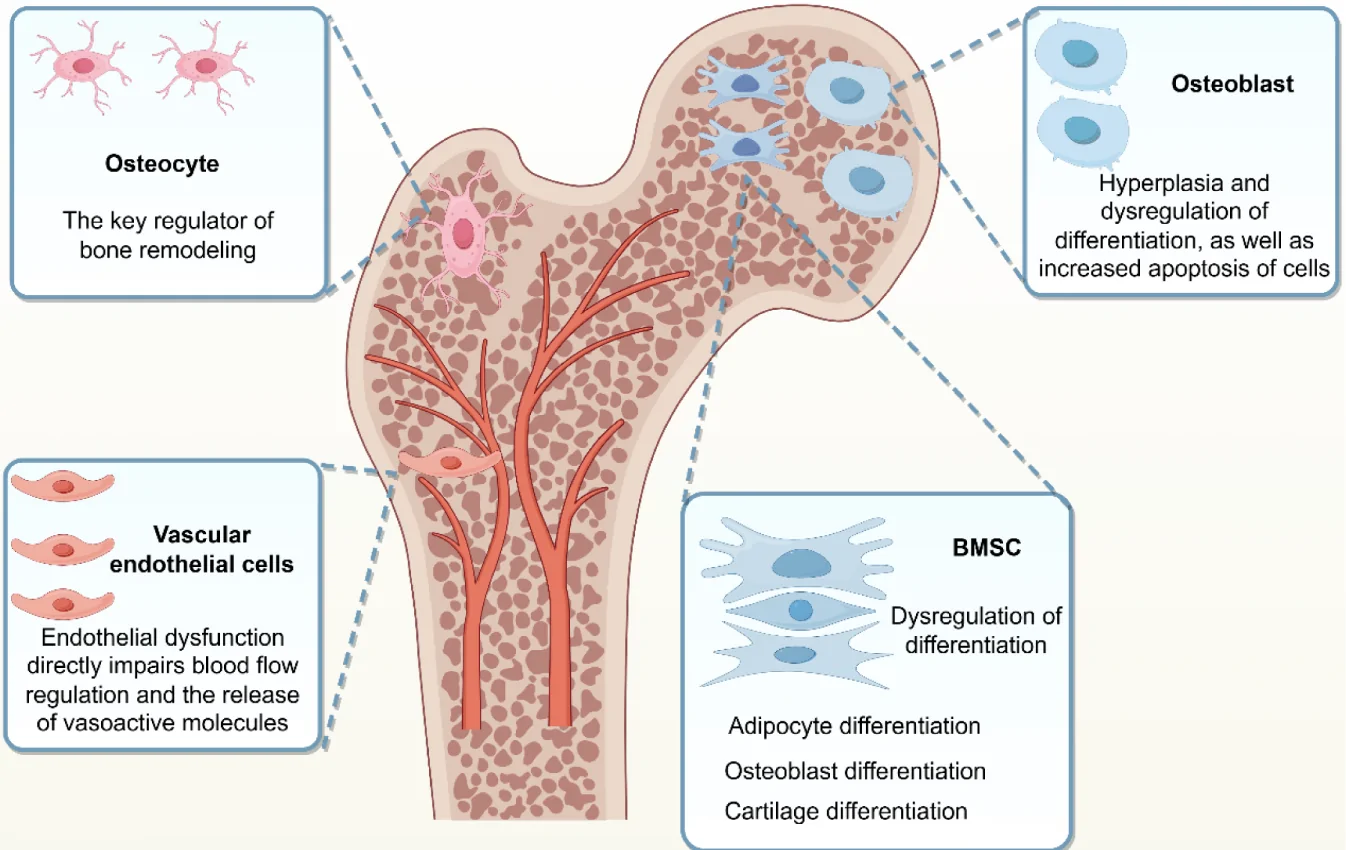
PTM-mediated regulation of key cellular fates in the initiation and progression of ONFH. Under pathogenic stimuli such as glucocorticoid exposure and alcohol consumption, aberrant post-translational modifications-including phosphorylation, acetylation/methylation, and ubiquitination-disrupt the osteogenic-adipogenic differentiation balance of BMSCs, induce apoptosis or ferroptosis in osteoblasts and osteocytes, and cause endothelial dysfunction and apoptosis. The integrated effects of these cellular events ultimately lead to impaired bone formation, excessive lipid accumulation, microvascular destruction, and local ischemia, which collectively drive the pathological progression of osteonecrosis of the femoral head.

In the context of ONFH pathogenesis, emerging evidence suggests that different types of PTMs contribute to disease progression by modulating multiple signaling pathways and triggering various forms of regulated cell death, including apoptosis, ferroptosis, and potentially other mechanisms (Yu et al., 2025), which are now recognized as critical processes in ONFH development. For instance, an imbalance between osteogenic and adipogenic differentiation of bone marrow-derived mesenchymal stem cells (BMSCs) is a key factor leading to impaired bone repair capacity, particularly in steroid-induced ONFH (Li X. et al., 2024). Excessive activation of osteoclasts accelerates bone resorption, disrupts bone remodeling homeostasis, and promotes structural deterioration of the femoral head (Vasiliadis et al., 2022). Osteocytes, serving as the primary mechanosensors within bone tissue, undergo apoptosis that not only compromises bone structural integrity but also aberrantly activates osteoclasts through the release of signaling molecules such as receptor activator of nuclear factor-κB ligand (RANKL), thereby establishing a vicious cycle (Jiang et al., 2021). In addition, endothelial cell dysfunction and apoptosis directly impair femoral head microcirculation, resulting in ischemic and hypoxic conditions that further exacerbate osteocyte death and tissue necrosis (Zhao et al., 2015). These complex intercellular interactions collectively constitute the pathological network of ONFH (Figure 1).

Common PTMs include phosphorylation, acetylation, methylation, ubiquitination, and others (Fries et al., 2017; Rhett et al., 2020; Xu and You, 2017). By regulating the proliferation, differentiation, apoptosis, and functional activity of key local cell populations in the femoral head-such as BMSCs, osteoblasts, osteocytes, and vascular endothelial cells-these PTMs actively participate in the initiation and progression of ONFH and represent core molecular mechanisms underlying cellular dysfunction (Yang et al., 2024; Lou et al., 2023; Duan et al., 2022). In this review, we systematically summarize the mechanisms by which different types of PTMs regulate the function of critical local cells in the femoral head and thereby influence ONFH pathogenesis. Furthermore, we discuss the potential of PTMs as early diagnostic biomarkers and therapeutic targets, and highlight future research directions, aiming to provide novel insights for the precise prevention and treatment of ONFH.

## 2 PTMs regulate ONFH progression by modulating BMSC fate

**FIGURE 2:**
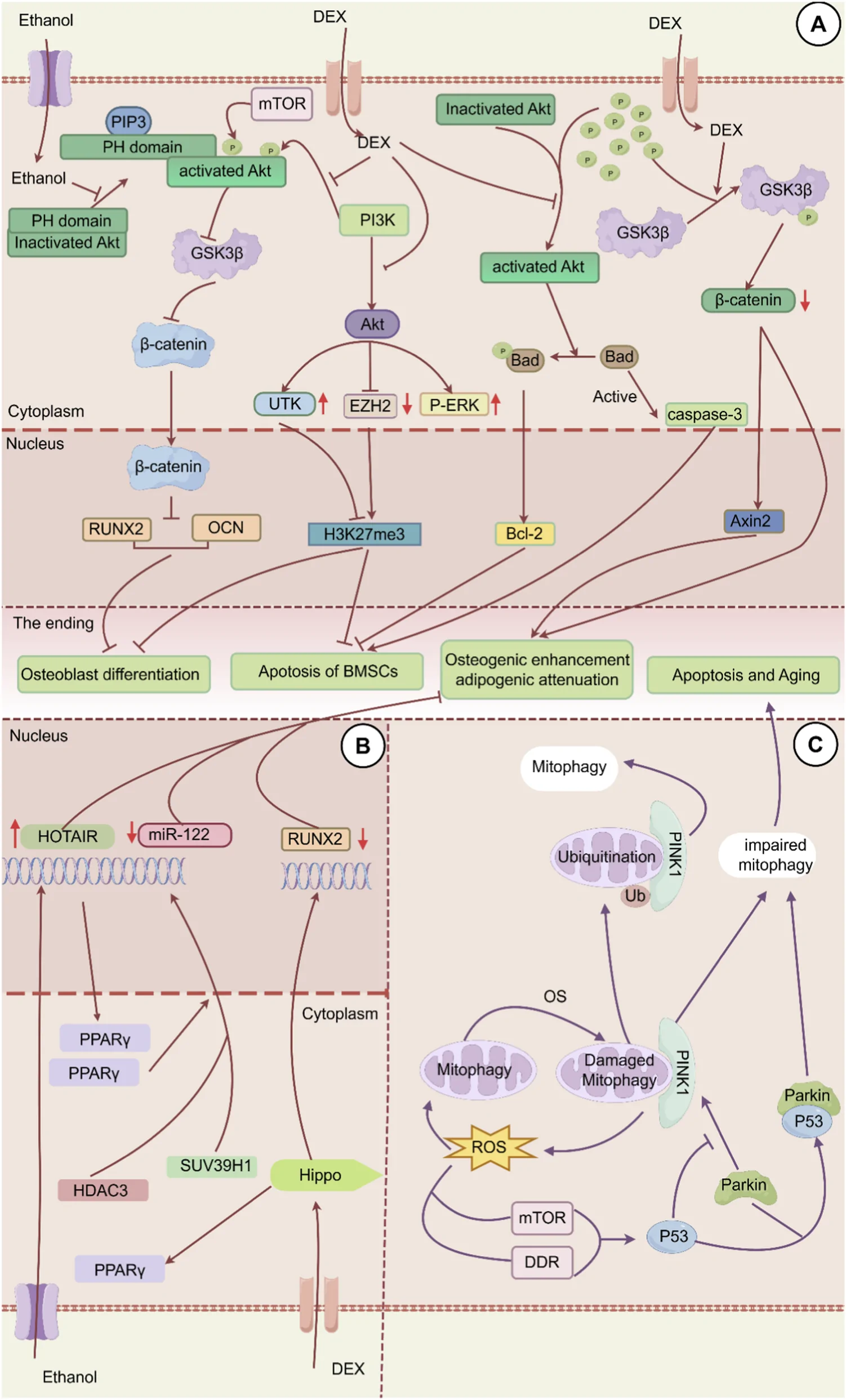
Molecular network of key PTM pathways regulating BMSC fate. **(A)** Phosphorylation-centered pathways, with Akt and GSK-3β/β-catenin as core nodes, regulate BMSC osteogenic differentiation and apoptosis in response to pathogenic stimuli. **(B)** Epigenetic reprogramming mediated by acetylation and methylation, together with lncRNA-driven dysregulation of histone modifications, promotes adipogenic lineage commitment at the expense of osteogenesis. **(C)** Ubiquitination mediated by E3 ligases such as NEDD4 and Parkin controls mitochondrial quality control and angiogenic signaling, thereby affecting BMSC survival and function. Collectively, these PTM networks underlie BMSC dysfunction in ONFH.

BMSCs are a critical population of adult stem cells within the femoral head microenvironment, possessing multilineage differentiation potential toward osteogenic, adipogenic, and chondrogenic lineages. Disruption of the osteogenic-adipogenic differentiation balance of BMSCs represents a central pathological event in ONFH, particularly in steroid-induced osteonecrosis of the femoral head (SONFH). Accumulating evidence indicates that PTMs profoundly influence the initiation and progression of ONFH by regulating BMSC fate decisions. Specifically, PTMs modulate osteogenic-adipogenic differentiation balance, apoptosis, and the activity of multiple signaling pathways in BMSCs, thereby exerting a decisive impact on disease progression (Figure 2) (Jia et al., 2023; Jiang et al., 2023).

### 2.1 Regulatory role of phosphorylation in BMSC fate determination

Protein phosphorylation is one of the most extensively studied enzymatic PTMs and is primarily catalyzed by protein kinases. During this process, phosphate groups derived from adenosine triphosphate (ATP) are covalently attached to specific amino acid residues of target proteins. Based on substrate specificity, protein kinases are classically categorized into serine (Ser), threonine (Thr), and tyrosine (Tyr) kinases. Although phosphorylation can also occur on residues such as cysteine, arginine, proline, aspartate, and histidine, Ser, Thr, and Tyr residues represent the predominant modification sites. It is important to distinguish protein kinases from phosphorylases, which utilize inorganic phosphate as a substrate and function through distinct catalytic mechanisms. Protein phosphorylation is dynamically and reversibly regulated by the coordinated actions of kinases and phosphatases within both cytoplasmic and nuclear compartments, serving as a fundamental mechanism for controlling protein activity, function, and signal transduction. Consequently, phosphorylation has emerged as one of the most intensively investigated PTMs (Wu et al., 2023).

#### 2.1.1 Akt-centered phosphorylation signaling in BMSC osteogenic dysfunction

In alcohol-induced ONFH, ethanol exposure impairs the recruitment of Akt to the plasma membrane and suppresses its phosphorylation at Ser473, thereby inhibiting the Akt/GSK-3β/β-catenin signaling cascade. This suppression leads to downregulation of osteogenic transcription factors, including RUNX2 and osteocalcin (OCN), ultimately inhibiting BMSC osteogenic differentiation (Figure 2A) (Chen et al., 2017).

Glucocorticoids (GCs), such as methylprednisolone and dexamethasone (DEX), exert profound effects on Akt phosphorylation and represent a key molecular mechanism underlying ONFH pathogenesis (Xu et al., 2021). DEX has been shown to inhibit the PI3K/Akt-UTX/EZH2 signaling axis by reducing PI3K and Akt phosphorylation levels, resulting in pathway inactivation. This suppression decreases the expression of UTX (a histone demethylase) while upregulating EZH2 (a histone methyltransferase), leading to enrichment of histone H3 lysine 27 trimethylation (H3K27me3), a canonical epigenetic marker of gene silencing. Consequently, osteogenesis-related genes are transcriptionally repressed, further exacerbating BMSC osteogenic inhibition (Ji et al., 2025).

Concurrently, DEX-induced apoptosis of BMSCs is closely associated with suppression of the Akt signaling pathway. The Akt/Bad/Bcl-2 axis is a critical regulator of cell survival, and inhibition of this pathway by DEX results in decreased Bcl-2 expression and increased caspase-3 activation, thereby aggravating BMSC apoptosis. This process compromises local bone repair capacity in the femoral head and promotes osteonecrosis development (Xu et al., 2025). Xu et al. (2021) demonstrated that overexpression of the long non-coding RNA LINC00473 upregulates phosphatidylethanolamine-binding protein 1 (PEBP1), thereby activating the Akt/Bad/Bcl-2 signaling pathway. This activation enhances Akt and Bad phosphorylation, increases anti-apoptotic Bcl-2 expression, and suppresses caspase-3 cleavage, highlighting a potential therapeutic target for SONFH (Figure 2A).

#### 2.1.2 Crosstalk between phosphorylation and epigenetic regulation

Icariin has been reported to activate the PI3K/Akt signaling pathway, upregulate UTX expression, and inhibit EZH2 activity, thereby reducing H3K27me3 enrichment. Through these mechanisms, icariin not only attenuates DEX-induced BMSC apoptosis but also promotes osteogenic differentiation-evidenced by increased expression of Bmp2 and Runx2-while suppressing adipogenic differentiation via downregulation of PPAR-γ and C/EBPα. In contrast, methylprednisolone (MPS) primarily disrupts the phosphorylation homeostasis of Akt, promoting BMSC apoptosis and indirectly accelerating femoral head necrosis (Jiang et al., 2025). Similarly, β-ecdysone has been shown to enhance Akt phosphorylation levels, thereby facilitating osteogenic differentiation of BMSCs (You and Xu, 2020).

#### 2.1.3 Phosphorylation-dependent regulation of wnt/β-catenin and TGF-β signaling

The Wnt/β-catenin signaling pathway functions as a pivotal “molecular switch” governing BMSC lineage commitment by promoting osteogenesis while suppressing adipogenesis. Steroids can activate glycogen synthase kinase 3β (GSK-3β) by increasing its phosphorylation at Tyr216, a modification associated with enhanced kinase activity. Activated GSK-3β promotes β-catenin phosphorylation and proteasomal degradation, resulting in reduced intracellular β-catenin levels and impaired transcriptional activity. This process amplifies the imbalance favoring adipogenesis over osteogenesis, ultimately driving ONFH progression (Yu et al., 2015). Crocin has been reported to promote osteogenic differentiation of BMSCs by suppressing GSK-3β phosphorylation (Li et al., 2020).

Recent studies have also identified DGCR5 as encoding a 102-amino acid peptide, RIP, which is highly expressed in BMSCs and femoral head tissues of SONFH patients. RIP binds to the N-terminal motif of RAC1, reduces RAC1-GTP levels, and suppresses the RAC1/PAK1 pathway. This inhibition decreases β-catenin phosphorylation at Ser675 and its nuclear translocation, leading to inactivation of the Wnt/β-catenin pathway. Consequently, BMSC differentiation is skewed toward adipogenesis while osteogenesis is suppressed, thereby aggravating SONFH progression (Jiang et al., 2023).

The transforming growth factor-β (TGF-β)/Smad signaling pathway is another essential regulator of BMSC osteogenic differentiation. Activation of this pathway promotes expression of the key osteogenic transcription factor Runx2 and facilitates bone mineralization. However, DEX significantly downregulates Runx2 mRNA expression, weakens the osteogenic capacity of BMSCs, and results in insufficient bone formation to repair femoral head tissue, ultimately leading to osteonecrosis (Fang et al., 2019).

Collectively, phosphorylation acts as a critical molecular switch in the regulation of BMSC fate. In response to steroid or alcohol exposure, alterations in the phosphorylation status of key signaling nodes-particularly Akt and GSK-3β/β-catenin-disrupt the balance between osteogenic and adipogenic differentiation and promote apoptotic signaling. Impaired Akt phosphorylation not only suppresses osteogenesis but also sensitizes BMSCs to apoptosis, underscoring the central role of phosphorylation-dependent mechanisms in BMSC dysfunction during ONFH pathogenesis (Figure 2A).

### 2.2 Coordinated regulation of acetylation and methylation in BMSC fate determination

Acetylation is a fundamental post-translational modification in which an acetyl group (-COCH_3_) is covalently transferred to lysine residues of histone or non-histone proteins in an enzyme-dependent manner. Based on the substrates involved, acetylation is broadly classified into histone acetylation and non-histone acetylation (Pai et al., 2024). Histone acetylation predominantly occurs at specific lysine residues located within the N-terminal tails of histones H3 and H4. This process is catalyzed by histone acetyltransferases (HATs), whereas the reverse reaction, deacetylation, is mediated by histone deacetylases (HDACs). By neutralizing the positive charge of lysine residues, histone acetylation relaxes chromatin structure and enhances DNA accessibility to transcription factors, thereby facilitating gene transcription. The dynamic balance between HAT and HDAC activities precisely regulates chromatin openness and transcriptional output (Hou et al., 2024).

Beyond histones, acetylation is widely observed in a broad spectrum of non-histone proteins, including transcription factors, metabolic enzymes, and cytoskeletal proteins. Non-histone acetylation modulates protein conformation, stability, and protein-protein interactions, thereby regulating diverse cellular processes at multiple levels. Importantly, this form of acetylation not only influences protein activity and subcellular localization but also participates in the regulation of cellular metabolism and signal transduction pathways (Ali et al., 2018).

Histone methylation represents another major epigenetic modification, typically occurring on lysine or arginine residues of histones. This modification is catalyzed by histone methyltransferases and reversed by specific demethylases. Unlike acetylation, histone methylation can exert either transcriptionally activating or repressive effects, depending on the specific residue and degree of methylation. Notably, acetylation and methylation frequently act in a coordinated manner, jointly modulating chromatin architecture and transcription factor activity to orchestrate gene expression programs.

#### 2.2.1 Acetylation-methylation crosstalk in alcohol- and steroid-induced ONFH

Ethanol exposure has been shown to upregulate the long non-coding RNA HOTAIR, leading to suppression of miR-122 expression and subsequent elevation of peroxisome proliferator-activated receptor γ (PPARγ) levels, thereby promoting adipogenic differentiation of BMSCs. Elevated PPARγ, in turn, recruits HDAC3 and the histone methyltransferase SUV39H1 to the miR-122 promoter region, inducing an imbalance between histone acetylation and methylation marks. This epigenetic remodeling results in sustained repression of miR-122, thereby perpetuating adipogenic programming even after alcohol withdrawal and contributing to the irreversible progression of ONFH (Le et al., 2023).

Similarly, CCAAT/enhancer-binding protein α (C/EBPα) has been reported to inhibit HDAC1 activity, leading to increased histone H3 lysine 27 acetylation (H3K27ac) at the PPARγ promoter region. Enhanced H3K27ac at the PPARγ locus constitutes a key epigenetic mechanism underlying steroid-associated osteonecrosis of the femoral head (SANFH). Importantly, pharmacological or genetic suppression of PPARγ-associated histone acetylation effectively inhibits adipogenic differentiation and attenuates SANFH progression (Duan et al., 2022).

In parallel, methylprednisolone has been shown to activate the Hippo signaling pathway, thereby suppressing osteogenic differentiation of BMSCs-as evidenced by downregulation of osteogenic markers such as RUNX2 and osteopontin-while simultaneously promoting adipogenic differentiation, characterized by increased triglyceride accumulation and upregulation of PPARγ and other adipogenic markers. This lineage shift results in reduced bone formation, excessive lipid accumulation within the marrow cavity, elevated intraosseous pressure, and exacerbated microvascular impairment, collectively accelerating ONFH progression (Sun et al., 2021).

#### 2.2.2 Epigenetic memory and sustained adipogenic commitment

Acetylation and methylation function as a dual epigenetic regulatory axis in ONFH, exhibiting pronounced cooperativity and persistence. Through coordinated modification of histone and non-histone proteins, these PTMs reshape the chromatin landscape and transcriptional programs, effectively “locking in” an adipogenic differentiation trajectory in BMSCs. Notably, even after removal of the initiating pathogenic stimuli, this epigenetic memory can persist, driving sustained adipogenic differentiation while repressing osteogenic and pro-survival gene expression. This phenomenon provides an epigenetic explanation for the progressive and often irreversible nature of ONFH and highlights acetylation-methylation crosstalk as a promising target for therapeutic intervention.

### 2.3 Regulatory role of ubiquitination in ONFH pathogenesis

Ubiquitination is a critical post-translational modification that involves the covalent attachment of ubiquitin molecules to specific residues of target proteins. This reversible process is catalyzed by E3 ubiquitin ligases, which confer substrate specificity and thereby precisely regulate protein stability, enzymatic activity, and protein-protein interaction networks (Hershko and Ciechanover, 1998; Zheng and Shabek, 2017; Kwon and Ciechanover, 2017). As a highly dynamic and multifunctional modification, ubiquitination participates in nearly all aspects of eukaryotic cellular physiology. Once the 76-amino acid ubiquitin protein is conjugated to a substrate, it can be further modified to generate diverse polyubiquitin chain architectures that elicit distinct cellular outcomes. This concept, known as the ‘ubiquitin code,’ represents a fundamental layer of post-translational regulation (Swatek and Komander, 2016). Through the coordinated actions of ubiquitin-activating enzymes (E1), ubiquitin-conjugating enzymes (E2), and ubiquitin ligases (E3), ubiquitination operates in a temporally and spatially controlled manner to fine-tune multiple cellular processes (Oikawa et al., 2023).

Within the ONFH microenvironment, oxidative stress (OS) plays a pivotal role in disrupting ubiquitin-dependent regulatory pathways. OS-induced upregulation of tumor suppressor protein p53 has been shown to inhibit the mitochondrial translocation of Parkin while simultaneously affecting its E3 ubiquitin ligase activity. This dysregulation impairs mitophagy, resulting in inefficient clearance of damaged mitochondria and subsequent accumulation of mitochondrial dysfunction. These findings provide novel mechanistic insights and potential therapeutic targets for early-stage steroid-induced ONFH (Figure 2B) (Zhang et al., 2020).

In addition to mitochondrial quality control, ubiquitination critically regulates angiogenesis-related signaling. Overexpression of the E3 ubiquitin ligase neural precursor cell expressed developmentally downregulated protein 4 (NEDD4) activates the vascular endothelial growth factor (VEGF) signaling pathway and cooperates with mammalian target of rapamycin (mTOR) signaling to enhance the viability, migratory capacity, and angiogenic potential of bone marrow endothelial cells (BMECs). However, glucocorticoids suppress NEDD4 expression by inducing hypermethylation of its promoter region, thereby impairing angiogenesis and exacerbating femoral head ischemia (Li et al., 2026).

Through precise control of protein turnover, mitochondrial quality control, and angiogenesis-related signaling, ubiquitination exerts a profound influence on BMSC survival and function. In ONFH, aberrant expression or activity of key E3 ubiquitin ligases, such as NEDD4 and Parkin, leads to defective mitophagy and impaired angiogenic responses, thereby aggravating BMSC dysfunction and failure of tissue repair. Dysregulation of the ubiquitin system not only reflects cellular homeostatic imbalance but also constitutes a self-amplifying feedback loop that drives sustained disease progression (Figure 2B).

## 3 PTMs modulate ONFH progression by regulating osteoblast function

Osteoblasts are the principal effector cells responsible for bone formation. Impaired proliferation and differentiation, together with increased cell death of osteoblasts, constitute key pathological mechanisms underlying reduced bone mass and compromised bone repair capacity in ONFH (Luo et al., 2024). PTMs participate in ONFH pathogenesis by modulating signaling pathway activity, mitochondrial function, and oxidative stress responses in osteoblasts.

### 3.1 Phosphorylation-dependent regulation of ferroptosis in osteoblasts

Osteoblasts are particularly vulnerable to GC-induced injury, and their loss constitutes a direct cause of failed bone formation in ONFH (Luo et al., 2024). While apoptosis has long been recognized as a major mechanism of osteoblast death, increasing evidence indicates that ferroptosis-a distinct form of regulated cell death characterized by iron dependency and lipid peroxidation-plays a critical role in GC-induced ONFH. Notably, the initiation and progression of ferroptosis are closely linked to phosphorylation-dependent signaling events.

Ferroptosis is driven by iron-catalyzed lipid peroxidation and is characterized by depletion of antioxidant molecules such as glutathione (GSH), reduced activity of antioxidant enzymes including superoxide dismutase 2 (SOD2) and glutathione peroxidase 4 (GPX4), and accumulation of oxidative damage markers such as malondialdehyde (MDA) (Zhao et al., 2025; Yuan et al., 2021). DEX has been shown to suppress the Janus kinase 2/signal transducer and activator of transcription 3 (JAK2/STAT3) signaling pathway by inhibiting phosphorylation of both JAK2 and STAT3. As a result, STAT3 fails to translocate into the nucleus and activate downstream transcriptional programs. This phosphorylation blockade leads to mitochondrial dysfunction and excessive generation of lipid reactive oxygen species (lipid ROS), ultimately triggering ferroptosis in osteoblasts (Lin et al., 2025).

**FIGURE 3:**
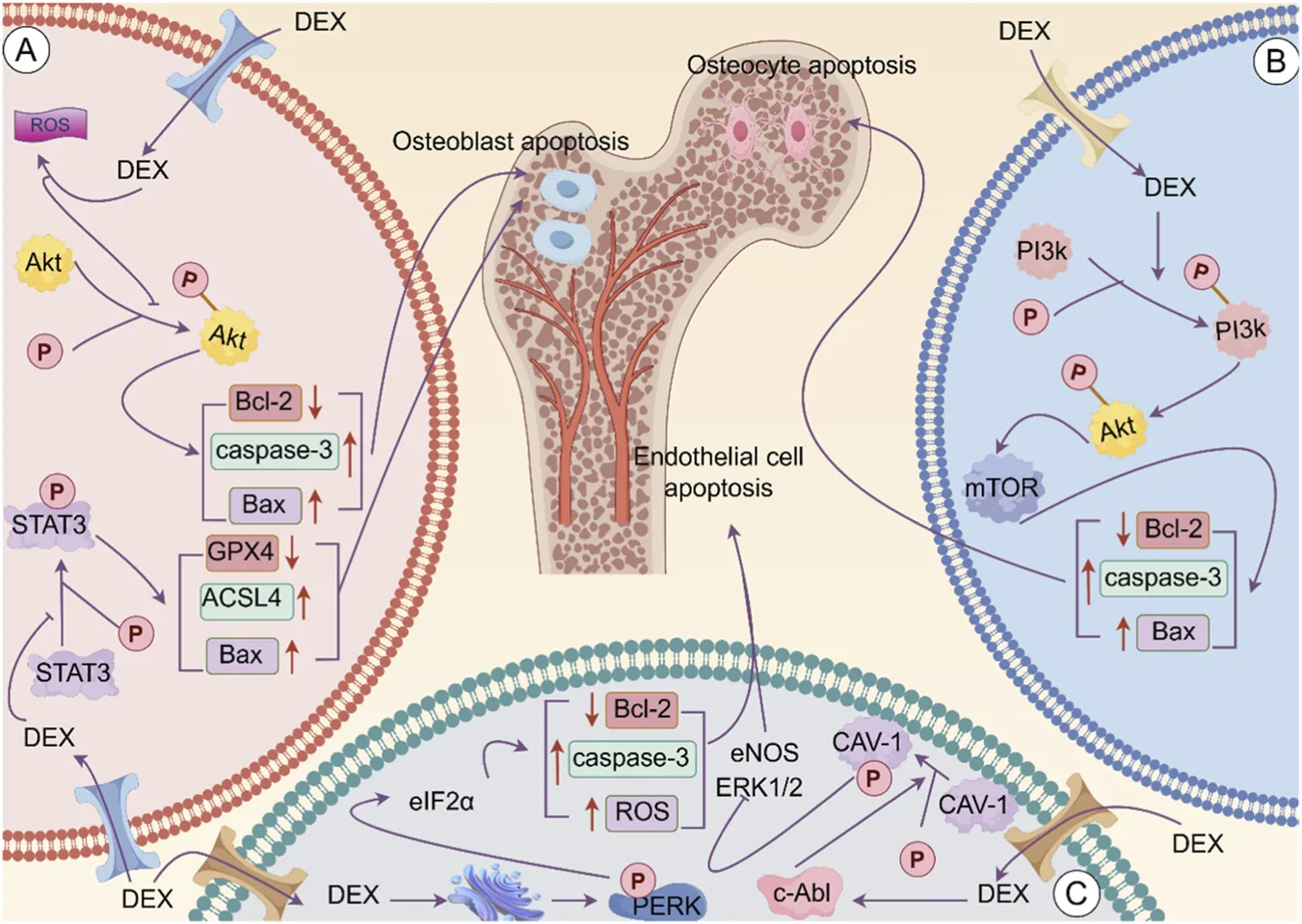
PTM-regulated cell death pathways in ONFH and potential therapeutic targets. This figure summarizes the critical roles of PTMs in regulating cell death pathways across different cell types involved in ONFH, including osteoblasts, osteocytes, and vascular endothelial cells. **(A)** GCs induce ferroptosis by inhibiting JAK2/STAT3 phosphorylation and promote apoptosis by disrupting PI3K/Akt and GSK-3β phosphorylation signaling. **(B)** DEX aberrantly activates the PI3K/Akt/mTOR pathway, leading to increased Bax and cleaved caspase-3 expression and reduced Bcl-2 levels, thereby enhancing osteoblast apoptosis. **(C)** In endothelial cells, DEX disrupts endoplasmic reticulum homeostasis and activates the PERK/p-eIF2α pathway, generating multiple pro-apoptotic signals (Bcl-2↓, cleaved caspase-3↑, ROS↑). In parallel, DEX activates c-Abl-mediated phosphorylation of CAV-1 (Y14), suppresses ERK1/2 and eNOS signaling, and promotes endothelial apoptosis. These events ultimately impair blood flow and contribute to the development of ONFH.

Concomitantly, DEX-induced ferroptosis is associated with downregulation of osteogenic markers, including collagen type I alpha 1 (COL1A1), RUNX2, and OCN. In parallel, antioxidant defenses are markedly compromised, as evidenced by reduced levels of GSH, SOD2, and GPX4, together with elevated MDA accumulation. These alterations synergistically exacerbate oxidative damage, impair osteoblast function, and compromise bone integrity, thereby accelerating ONFH progression (Figure 3A) (Lin et al., 2025).

### 3.2 Phosphorylation-mediated mitochondrial apoptosis in osteoblasts

In addition to ferroptosis, phosphorylation-dependent regulation of mitochondrial apoptosis represents another major mechanism contributing to osteoblast loss in ONFH. The phosphoinositide 3-kinase (PI3K)/Akt pathway is a central signaling axis for maintaining osteoblast survival. Glucocorticoids suppress this pathway by reducing Akt phosphorylation, leading to downregulation of the anti-apoptotic protein Bcl-2 and upregulation of pro-apoptotic Bax and executioner cleaved caspase-3. The resulting increase in the Bax/Bcl-2 ratio activates the caspase cascade, thereby promoting osteoblast apoptosis (Deng et al., 2018).

Conversely, sphingosine-1-phosphate (S1P) has been reported to attenuate DEX-induced osteoblast injury by restoring Akt phosphorylation, highlighting the protective role of Akt-centered phosphorylation signaling in osteoblast survival (Ji et al., 2015). In parallel, GCs reduce phosphorylation of glycogen synthase kinase 3β (GSK3β) at Ser9, resulting in GSK3β activation. Activated GSK3β triggers mitochondrial apoptotic signaling, characterized by cytochrome c release and subsequent activation of caspase-9 and caspase-3, ultimately leading to osteoblast apoptosis, bone loss, and ONFH development (Nie et al., 2019).

Collectively, osteoblast loss represents a direct determinant of impaired bone formation in ONFH. Beyond classical apoptosis, ferroptosis has emerged as a critical mechanism of glucocorticoid-induced osteoblast injury. Phosphorylation-dependent modulation of key signaling pathways, including JAK2/STAT3 and PI3K/Akt/GSK3β, coordinately regulates both ferroptotic and apoptotic processes. By suppressing kinase phosphorylation, glucocorticoids induce mitochondrial dysfunction, lipid peroxidation, and multiple forms of regulated cell death. These findings underscore the central role of PTMs as integrative regulators of osteoblast fate and highlight their potential as therapeutic targets in ONFH.

## 4 PTMs regulate ONFH progression by modulating endothelial cell function

Endothelial cells are essential for maintaining vascular integrity, regulating blood flow, and preserving endothelial homeostasis (Zhang et al., 2011; Dai et al., 2020). Endothelial dysfunction directly disrupts vascular tone regulation and the release of vasoactive mediators, thereby disturbing the delicate balance between vasoconstriction and vasodilation (Li et al., 2025). As a consequence, local blood supply becomes insufficient to meet the metabolic demands of bone tissue, ultimately leading to ischemic injury.

Notably, endothelial damage alters intravascular hemodynamics, promoting a transition from laminar to turbulent blood flow. This hemodynamic disturbance facilitates thrombosis formation and delays fibrinolysis within thrombi, further compromising vascular patency and exacerbating ischemia (Kakar and Lip, 2007).Sustained hypoxic stress in bone tissue subsequently induces mitochondrial calcium overload, mitochondrial fragmentation, and osteocyte apoptosis, thereby accelerating femoral head collapse (Xu et al., 2024).

GCs exert profound effects on endothelial cell survival through phosphorylation-dependent stress signaling. Specifically, GCs activate the protein kinase R-like endoplasmic reticulum kinase (PERK)/eukaryotic initiation factor 2α (eIF2α) phosphorylation cascade in vascular endothelial cells, ultimately triggering C/EBP homologous protein (CHOP)-mediated apoptosis. CHOP not only directly or indirectly suppresses the expression of the anti-apoptotic protein Bcl-2 but also promotes the activation of cleaved caspase-3 and the upregulation of the pro-apoptotic protein Bax. In parallel, CHOP signaling enhances intracellular reactive oxygen species (ROS) accumulation, further amplifying oxidative stress and endothelial apoptosis (Sun et al., 2024).

In steroid-associated osteonecrosis of the femoral head, GC signaling through the glucocorticoid receptor (GR) activates c-Abl kinase, leading to phosphorylation of caveolin-1 (CAV-1) and subsequent endothelial dysfunction. Beyond this direct endothelial effect, GCs also suppress sympathetic neural outflow from the paraventricular nucleus via GR-dependent mechanisms, resulting in reduced norepinephrine release. This neurovascular alteration downregulates the expression of the glycolysis-associated enzyme 6-phosphofructo-2-kinase/fructose-2,6-bisphosphatase 3 (PFKFB3) in endothelial cells, thereby impairing endothelial glycolysis and inhibiting angiogenesis-osteogenesis coupling (Figure 3B) (Fang et al., 2025).

Endothelial apoptosis represents an initiating event in microvascular destruction and ischemia during ONFH development. PTMs, particularly phosphorylation and ubiquitination, regulate key signaling pathways such as PERK/eIF2α and c-Abl/CAV-1, thereby controlling endothelial cell survival, vascular tone, and neovascularization. Targeting these PTM-regulated nodes may provide a promising strategy to preserve intraosseous blood supply at an early stage and interrupt the vicious cycle of ischemia and necrosis in ONFH.

## 5 PTMs regulate ONFH progression by modulating osteoclast formation and bone resorption

Osteoclasts are the primary bone-resorbing cells responsible for maintaining skeletal remodeling, and excessive osteoclast activation is recognized as a major contributor to subchondral bone destruction during ONFH (Yari et al., 2023). GC exposure induces excessive intracellular accumulation of ROS, generating oxidative stress that serves as an upstream trigger for phosphorylation-dependent signaling cascades governing osteoclast differentiation (Chen et al., 2020). Specifically, GC-induced oxidative stress promotes the phosphorylation and activation of TRAF6, which subsequently stimulates downstream JNK and NF-κB signaling pathways. Activated JNK and NF-κB translocate into the nucleus and upregulate key osteoclastogenic transcription factors, including NFATc1 and c-Fos, thereby accelerating the proliferation, fusion, and maturation of osteoclast precursors. Mature osteoclasts secrete large amounts of matrix metalloproteinases and hydrogen ions, leading to degradation of the trabecular bone matrix and disruption of physiological bone remodeling homeostasis. This imbalance, characterized by impaired osteogenesis and excessive bone resorption, exacerbates trabecular thinning and microfracture formation, ultimately resulting in irreversible femoral head collapse (Yi et al., 2019; Balogh et al., 2018).

Aberrant phosphorylation of the TRAF6/JNK/NF-κB signaling axis represents a critical post-translational modification event underlying GC-induced osteoclast hyperactivation. Targeting this phosphorylation cascade has emerged as a promising strategy for preventing pathological bone loss. Notably, Resina Draconis has been shown to attenuate ROS accumulation and suppress TRAF6/JNK pathway-mediated osteoclast activation, thereby alleviating osteonecrotic lesions and slowing disease progression in ONFH (Liu et al., 2023).

## 6 PTMs influence ONFH progression by regulating osteocyte survival and function

Osteocytes, the most abundant and longest-living cells in bone tissue, serve as central regulators of bone remodeling and skeletal homeostasis (Prideaux et al., 2015). The survival and functional integrity of osteocytes are essential for maintaining bone structure and mechanical competence. Any factor that compromises osteocyte viability may disrupt bone homeostasis and predispose the skeleton to pathological remodeling, ultimately contributing to bone-related diseases (Tao H. et al., 2024).

Osteocyte apoptosis represents a critical event in the pathogenesis of ONFH. It is widely recognized as an early trigger for osteoclast recruitment and excessive bone resorption, thereby accelerating structural deterioration of the femoral head. Among the signaling pathways governing osteocyte survival, the PI3K/AKT/mTOR axis plays a dominant role. Abnormal activation of this pathway, reflected by elevated phosphorylation levels in the femoral heads of steroid-induced ONFH rat models, has emerged as a noteworthy phenomenon warranting further investigation.

A prevailing hypothesis suggests that sustained hyperactivation of the PI3K/AKT/mTOR pathway may initially represent a compensatory pro-survival response to ischemic or glucocorticoid-induced stress. However, prolonged or dysregulated phosphorylation signaling may ultimately lead to feedback inhibition, metabolic imbalance, or cellular stress overload, thereby rendering osteocytes more susceptible to apoptosis (Su and Chen, 2025). This shift from adaptive survival signaling to maladaptive cell death underscores the dual and context-dependent nature of phosphorylation-mediated regulation in osteocytes (Figure 3C).

As key orchestrators of bone homeostasis, osteocytes occupy a pivotal position in the progression of ONFH. Aberrant phosphorylation states of survival-related pathways, such as PI3K/AKT/mTOR, may drive osteocytes to transition from stress adaptation toward apoptosis, thereby amplifying bone resorption and structural collapse. Although current studies on PTMs in osteocytes remain limited, their numerical dominance and longevity within bone tissue suggest that osteocyte-centered PTM regulatory networks are likely to play a crucial role in intercellular communication and systemic imbalance during ONFH progression. Elucidating these mechanisms may provide novel insights into disease pathogenesis and uncover new therapeutic targets.

To more systematically illustrate the regulatory roles of different protein post-translational modifications in key cellular populations involved in ONFH, the table below summarizes how major PTM types-including phosphorylation, acetylation, methylation, and ubiquitination-contribute to the initiation and progression of ONFH by modulating signaling pathways and functional states of BMSCs, osteoblasts, osteocytes, and vascular endothelial cells. The table is organized across three dimensions-target cell type, modification type, and underlying mechanism-and is intended to provide a clear, structured overview of the multidimensional PTM-mediated regulatory network in ONFH. This integrative framework facilitates a deeper understanding of PTM-driven cellular dysregulation and highlights potential molecular nodes for diagnostic and therapeutic intervention (Table1).

**TABLE 1:** Roles of different PTM types in distinct cell populations contributing to the initiation and progression of ONFH.

Target cell type	PTM type	Mechanism	References
BMSC	Phosphorylation	Ethanol specifically inhibits Akt phosphorylation at Ser473, resulting in reduced Akt activity and suppression of the Akt/GSK3β/β-catenin pathway, thereby decreasing osteogenic markers such as COL1, OPN, and OCN.	Chen et al. (2017)
Osteoblast	Phosphorylation	Ischemia/hypoxia induces endoplasmic reticulum stress, activating PERK and phosphorylating eIF2α. p-eIF2α suppresses global protein translation while selectively enhancing ATF4 translation, upregulating CHOP and promoting osteoblast apoptosis	Liu et al. (2017)
BMSC	Phosphorylation and ubiquitination	GC exposure markedly reduces USP13 expression. USP13 deubiquitinates and stabilizes PTEN, thereby inhibiting PI3K/Akt signaling and exerting anti-apoptotic effects	Jiang et al. (2025)
BMSC	Phosphorylation	Glucocorticoids activate the negative regulator GSK-3β, suppressing Wnt/β-catenin signaling and inhibiting osteogenic differentiation of BMSCs	Li et al. (2020)
BMSC	Phosphorylation	DEX activates caspase-3 and caspase-9 pathways, increases Bax and decreases Bcl-2 expression, resulting in reduced BMSC survival and disruption of the bone marrow hematopoietic microenvironment	Ji et al. (2025), You and Xu (2020)
BMSC	Phosphorylation	In ONFH models, aberrant activation of the PI3K/Akt pathway leads to elevated Akt phosphorylation, which paradoxically suppresses osteogenic differentiation of BMSCs	You and Xu (2020)
Endothelial cell	Phosphorylation	GC indirectly inhibits Akt phosphorylation, impairing its anti-apoptotic, pro-angiogenic, and pro-osteogenic effects and exacerbating ER stress-mediated endothelial dysfunction	Sun et al. (2024)
Endothelial cell	Phosphorylation	GCs markedly suppress PI3K and Akt phosphorylation in BMECs, leading to pathway inactivation, reduced angiogenesis, and enhanced endothelial injury	Huang et al. (2024b)
Endothelial cell	Phosphorylation	GCs inhibit Akt phosphorylation, thereby reducing HIF-1α expression and nuclear translocation, decreasing VEGF secretion and impairing endothelial proliferation, migration, and tube formation	Shan et al. (2023)
Osteoblast	Phosphorylation	GCs suppress phosphorylation of JAK2 and STAT3, leading to pathway inactivation, downregulation of anti-ferroptotic molecules (GPX4, SOD2) and osteogenic genes (COL1A1, RUNX2, OCN), thereby promoting osteoblast ferroptosis and impairing bone formation	Lin et al. (2025)
Osteoblast	Phosphorylation	GC exposure suppresses GSK-3β Ser9 phosphorylation, activating the mitochondrial apoptotic pathway (mitochondrial depolarization, cytochrome c release, caspase cascade), resulting in increased osteoblast apoptosis	Nie et al. (2019)
Osteoblast	Phosphorylation	GCs significantly reduce p-PI3K, p-Akt, and p-GSK3β levels, leading to pathway inactivation and loss of anti-apoptotic, pro-proliferative, and osteogenic functions	Xuecheng et al. (2025), Xue et al. (2018)
Osteoblast	Phosphorylation	GC exposure activates and induces nuclear translocation of TAT1, which regulates the mitochondrial apoptotic pathway (Bax/Bcl-2 imbalance, cytochrome c release), triggering caspase-9/3 activation and osteoblast apoptosis	Feng et al. (2017)
Osteoclast	Phosphorylation	GC-induced ROS accumulation activates TRAF6/JNK and NF-κB signaling, promoting abnormal osteoclast proliferation, differentiation, and bone-resorptive activity	Liu et al. (2023)
Endothelial cell	Phosphorylation	GCs bind GR to activate c-Abl, inducing aberrant CAV-1 phosphorylation, suppressing ERK1/2 and eNOS signaling, and increasing endothelial apoptosis	Fang et al. (2025)
Osteocyte	Phosphorylation	GCs markedly increase p-PI3K, p-Akt, and p-mTOR levels in femoral head tissue, leading to pathway overactivation and triggering mitochondrial apoptosis (Bax↑, cleaved caspase-3↑, Bcl-2↓)	Su and Chen (2025)
Osteoblast	Ubiquitination	GC-induced downregulation of miR-3175 and upregulation of DCAF1 enhance Nrf2 degradation, impair antioxidant signaling, increase oxidative stress, and promote osteoblast apoptosis	Chen et al. (2021)
Endothelial cell	Ubiquitination	GC increases NEDD4 promoter methylation, suppressing NEDD4 expression and impairing VEGF signaling and mTOR cooperation, resulting in defective angiogenesis	Li et al. (2026)
BMSC	Ubiquitination	Oxidative stress in necrotic regions upregulates p53, which binds Parkin and inhibits PINK1-Parkin-mediated mitophagy, leading to mitochondrial dysfunction, ROS accumulation, and BMSC apoptosis or senescence	Zhang et al. (2020)
Osteocyte	Ubiquitination	GC suppresses TRIM21 expression, disrupting NF-κB/IRF3/7 signaling and causing excessive IFN-α accumulation, ultimately inducing osteocyte and bone marrow cell apoptosis	Tateda et al. (2012)
Osteocyte	Acetylation	GC downregulates HDAC1, leading to excessive SP1 acetylation and accumulation, upregulation of pro-apoptotic proteins (Bax, cleaved caspase-3), increased osteocyte apoptosis, and reduced proliferation	Zhang et al. (2025)
Osteoblast	Acetylation	GC exposure reduces cPWWP2A expression and causes abnormal accumulation of miR-579, which targets and suppresses SIRT1 and PDK1, leading to insufficient Akt activation and osteoblast apoptosis or programmed necrosis	Hong et al. (2019b)
Endothelial cell	Acetylation	GC disrupts the NAMPT/STK11/HMGCR/ACAT1 axis, resulting in lipid metabolic disorders, inflammatory activation, hypercoagulability, and bone metabolic imbalance	Wu et al. (2025)
BMSC	Methylation and acetylation	Chronic alcohol exposure upregulates Lnc-HOTAIR, sponges miR-122, and increases PPARγ expression, promoting adipogenic differentiation, suppressing osteogenesis, increasing marrow fat accumulation and intraosseous pressure, and impairing local blood supply	Le et al. (2023)
BMSC	Acetylation	GC-GR binding upregulates C/EBPα and suppresses HDAC1, increasing H3K27ac levels and sustaining PPARγ activation, thereby inhibiting osteogenesis and enhancing adipogenesis of BMSCs	Duan et al. (2022)

## 7 Small-molecule therapeutics targeting PTMs: a novel direction for ONFH treatment

PTMs act as central molecular switches that govern cell fate decisions, thereby offering a fundamentally new therapeutic perspective for ONFH. With the growing understanding of key PTM events and their functional consequences during ONFH pathogenesis, the development of PTM-related signaling modulators capable of selectively modulating these modifications has emerged as a highly promising treatment strategy. Notably, these compounds indirectly alter cellular PTM profiles through upstream or downstream signal transduction, rather than directly acting on PTM-modifying enzymes such as kinases and deacetylases.

The pathological core of ONFH lies in the dysfunction and death of critical cellular populations within the femoral head, including BMSCs, osteoblasts, osteocytes, and vascular endothelial cells. PTMs-such as phosphorylation, acetylation/methylation, and ubiquitination-precisely regulate intracellular signaling cascades, transcriptional programs, protein stability, and organelle homeostasis. Through these mechanisms, PTMs directly determine key cellular outcomes, including survival, differentiation, apoptosis, and ferroptosis. All compounds listed in Table 2 belong to PTM-related signaling modulators, which exert indirect regulatory effects on PTM status instead of directly modifying catalytic enzymes for post-translational modifications.

**TABLE 2:** PTM-related signaling modulators: Small-molecule agents that regulate PTM-dependent signaling pathways to delay or prevent the initiation and progression of ONFH.

Small-molecule drug	PTM	Mechanism	Target cell	References
Ortho-silicic acid (OSA)	Phosphorylation	OSA increases Akt phosphorylation (p-Akt), alleviates GC-induced endoplasmic reticulum stress, enhances endothelial cell function and osteogenic differentiation, and thereby attenuates ONFH progression	Endothelial cells	Sun et al. (2024)
Astaxanthin	Phosphorylation	Astaxanthin activates the JAK2/STAT3 signaling pathway, upregulates anti-ferroptotic molecules, suppresses osteoblast ferroptosis, and protects against ONFH.	Osteoblasts	Lin et al. (2025)
Selenium (Se)	Phosphorylation	Selenium activates the PI3K/AKT/GSK3β signaling axis, restores osteoblast survival and function, and prevents GC-induced ONFH.	Osteoblasts	Xuecheng et al. (2025)
Fludarabine	Phosphorylation	Fludarabine inhibits STAT1-dependent caspase-3 upregulation, thereby suppressing osteoblast apoptosis	Osteoblasts	Feng et al. (2017)
Pravastatin	Phosphorylation	Pravastatin induces AMPK phosphorylation and suppresses mTOR activity, activates autophagy in endothelial cells, improves microcirculation, and alleviates ischemic ONFH.	Endothelial cells	Liao et al. (2018)
Dragon’s blood resin (DBR)	Phosphorylation	DBR suppresses ROS accumulation and inhibits osteoclast activation, thereby reducing bone resorption and ONFH severity	Osteoclasts	Liu et al. (2023)
Salidroside	Phosphorylation	Salidroside activates the PI3K/Akt pathway and downregulates caspase-3 expression, attenuating Dex-induced osteoblast apoptosis	Osteoblasts	Xue et al. (2018)
Irisin	Phosphorylation	Irisin inhibits GC-induced c-Abl and CAV-1 phosphorylation via integrin αVβ5, preserves ERK and eNOS signaling, and protects endothelial angiogenic and migratory functions	Endothelial cells	Fang et al. (2025)
Tanshinone I	Phosphorylation	Tanshinone I suppresses aberrant activation of the PI3K/AKT/mTOR pathway, thereby limiting osteocyte apoptosis	Osteocytes	Su and Chen (2025)
Sesamin	Phosphorylation	Sesamin reduces ROS accumulation, suppresses mitochondria-dependent osteoblast apoptosis, and protects against femoral head osteonecrosis	Osteoblasts	Deng et al. (2018)
Icariin	Phosphorylation	Icariin activates the PI3K-AKT-UTX/EZH2 signaling axis, promotes osteogenic differentiation of BMSCs, and counteracts steroid-induced ONFH.	BMSCs	Ji et al. (2025)
Astragaloside IV	Phosphorylation	Astragaloside IV promotes osteogenesis and angiogenesis via Akt/Runx2 and Akt/HIF-1α/VEGF pathways, respectively, while inhibiting apoptosis and oxidative stress via Akt/Bad/Bcl-2 and Akt/Nrf2/HO-1 signaling	Endothelial cells	Shan et al. (2023)
Crocin	Phosphorylation	Crocin suppresses GSK-3β phosphorylation, accelerates osteogenic differentiation of BMSCs, and facilitates recovery from SANFH.	BMSCs	Li et al. (2020)
Lithium chloride (LiCl)	Phosphorylation	LiCl activates the β-catenin signaling pathway, inhibits aberrant adipogenesis, and enhances osteogenic differentiation of ONFH-derived BMSCs	BMSCs	Yu et al. (2015)
β-ecdysone	Phosphorylation	β-ecdysone enhances Akt phosphorylation, promotes osteogenic differentiation of BMSCs, and contributes to functional recovery in ONFH.	BMSCs	You and Xu (2020)

Aberrant PTM patterns induced by glucocorticoid exposure, ischemia, oxidative stress, or metabolic imbalance disrupt the coordinated function of osteogenic and angiogenic systems, ultimately leading to impaired bone repair and progressive femoral head collapse. In this context, PTM-related signaling modulators that intervene upstream and downstream signaling axes to reverse pathological PTM abnormalities-by inhibiting or activating specific kinases, deacetylases, methyltransferases, or E3 ubiquitin ligases-hold the potential to restore cellular homeostasis at its molecular origin.

By correcting abnormal PTM-driven signaling rather than merely alleviating downstream symptoms, these PTM-related signaling modulators may reestablish osteogenic capacity, preserve vascular integrity, and enhance bone-vascular coupling. Consequently, such therapeutic strategies could delay disease progression, improve structural preservation of the femoral head, and potentially reverse early-stage ONFH. Collectively, intervening PTM-associated signaling cascades via small-molecule modulators represents a mechanism-oriented and highly translational therapeutic avenue, opening new possibilities for precision intervention in ONFH (Table 2).

## 8 Perspectives and future

Although substantial progress has been made in elucidating the roles of PTMs in ONFH, this field remains at an early developmental stage and is characterized by both significant challenges and emerging opportunities. A central task for future research is to translate mechanistic insights from basic studies into clinically actionable diagnostic and therapeutic strategies. At present, early diagnosis of ONFH remains difficult. Future investigations should leverage high-throughput proteomic approaches, particularly mass spectrometry-based analyses, to systematically profile and identify disease-specific PTM signatures in serum, synovial fluid, or tissue samples from ONFH patients. Distinct phosphorylation, acetylation, or ubiquitination patterns of specific proteins may serve as sensitive and specific noninvasive or minimally invasive biomarkers for early detection, risk stratification, and prognostic assessment. Nevertheless, the therapeutic exploitation of PTM-associated signaling pathways is complicated by their context-dependent biological effects. A representative example is the PI3K/Akt pathway, which may exert distinct functions across different cell populations and stages of ONFH progression. In osteogenic precursor cells, including BMSCs and osteoblasts, glucocorticoid-induced suppression of PI3K/Akt signaling impairs osteogenic differentiation and promotes apoptosis, whereas restoration of Akt phosphorylation generally confers cytoprotective and pro-osteogenic effects. In contrast, in osteocytes exposed to prolonged glucocorticoid and ischemic stress, sustained activation of the PI3K/Akt/mTOR axis has been associated with defective mitophagy, oxidative stress accumulation, and cell death. These findings suggest that the biological consequences of PTM-regulated signaling are highly dependent on cellular context, disease stage, and stimulus intensity. Therefore, future PTM-targeted therapeutic strategies should prioritize cell type-specific and temporally controlled modulation rather than indiscriminate activation or inhibition of individual signaling pathways.

To date, the development of PTM-targeting drugs has largely been confined to the oncology field (Li QQ. et al., 2017; Liu et al., 2024). In the context of ONFH, the rational design of small-molecule inhibitors or activators that selectively modulate key PTM-regulating enzymes-such as kinases, deacetylases, or E3 ubiquitin ligases-represents a highly promising therapeutic avenue. For example, agents that enhance Akt phosphorylation at specific sites or inhibit GSK3β activity may help restore the osteogenic differentiation capacity of BMSCs. However, achieving cell type- and pathway-specific modulation remains a major challenge to minimize off-target effects. In this regard, nanocarrier-based delivery systems offer a viable strategy to enable targeted drug delivery, thereby improving therapeutic efficacy while reducing systemic toxicity.

Proteins are typically subject to multiple PTMs simultaneously, forming a complex and dynamic “PTM code” (Kay et al., 2024; Haj-Yahya and Lashuel, 2018). Future studies must therefore move beyond a single-PTM perspective to investigate the crosstalk and combinatorial effects among different PTMs. Traditional cell and animal models are often insufficient to fully recapitulate the complexity of the human bone microenvironment. The establishment of patient-derived femoral head organoid models, generated from stem cells, may provide a more faithful representation of ONFH pathology and serve as powerful platforms for high-throughput drug screening and mechanistic studies. Moreover, the integration of multi-omics datasets-including proteomics, epigenomics, and transcriptomics-combined with artificial intelligence and machine learning approaches, holds promise for constructing predictive models capable of forecasting disease progression and guiding personalized therapeutic strategies.

Beyond classical PTMs such as phosphorylation and acetylation, emerging metabolism-related modifications, including lactylation and succinylation, are attracting increasing attention. Given that metabolic disturbances-such as alcohol abuse and glucocorticoid exposure-are major risk factors for ONFH (Martiniakova et al., 2018; Kamal et al., 2012), these novel PTMs that directly link cellular metabolism to epigenetic regulation may play previously unrecognized but critical roles in disease pathogenesis.

Collectively, PTMs function as molecular switches governing the fate of key cell populations in ONFH. Future research should prioritize the systematic mapping of cell type-specific PTM codes under ONFH-inducing conditions. Such efforts will clarify how combinatorial PTMs determine the balance between cell survival and death. In particular, elucidating the crosstalk between phosphorylation and acetylation in regulating key ferroptosis-related factors, such as GPX4, will be of critical importance. PTM-based therapeutic development should focus on agents capable of simultaneously enhancing pro-survival pathways (e.g., Akt phosphorylation) while suppressing pro-death signaling cascades (e.g., JAK2/STAT3-mediated ferroptosis). Patient-derived ONFH organoid models will be invaluable for validating the functional relevance of specific PTM events in human cell death processes and for high-throughput screening of PTM-modulating compounds.

Despite growing recognition of the importance of PTMs in cellular homeostasis, existing studies have largely focused on isolated PTMs (e.g., phosphorylation) within single cell types (e.g., BMSCs). A comprehensive framework that integrates how multiple key PTMs cooperatively regulate the fate of all major cellular constituents of the femoral head, ultimately converging on the common endpoint of osteonecrosis, is still lacking. In particular, the precise mechanisms by which PTMs act as molecular hubs linking exogenous pathogenic stimuli, such as glucocorticoids, to intracellular programs of regulated cell death-including apoptosis and ferroptosis-remain to be fully elucidated.
